# Conservation genomics within government led conservation planning: an Australian case study exploring cost and benefit for threatened flora

**DOI:** 10.1093/aob/mcae222

**Published:** 2025-07-14

**Authors:** Chantelle A T Doyle, Manuela Cascini, Jia-Yee Samantha Yap, Hannah Matthews, Patricia M Hogbin, Trevor C Wilson, Erica Mahon, Dianne Brown, Aaron Mulcahy, Rachel Brown, Maurizio Rossetto

**Affiliations:** Research Centre for Ecosystem Resilience, Botanic Gardens of Sydney, Sydney, NSW, 2000, Australia; Centre for Ecosystem Science, University of New South Wales, Kensington, NSW, 2052, Australia; Research Centre for Ecosystem Resilience, Botanic Gardens of Sydney, Sydney, NSW, 2000, Australia; Research Centre for Ecosystem Resilience, Botanic Gardens of Sydney, Sydney, NSW, 2000, Australia; NSW Department of Climate Change, Energy, the Environment and Water – Biodiversity, Conservation and Science, Parramatta, NSW, 2150, Australia; Research Centre for Ecosystem Resilience, Botanic Gardens of Sydney, Sydney, NSW, 2000, Australia; Plant Discovery and Evolution, Botanic Gardens of Sydney, Mount Annan, NSW, 2567, Australia; NSW Department of Climate Change, Energy, the Environment and Water – Biodiversity, Conservation and Science, Parramatta, NSW, 2150, Australia; NSW Department of Climate Change, Energy, the Environment and Water – Biodiversity, Conservation and Science, Parramatta, NSW, 2150, Australia; NSW Department of Climate Change, Energy, the Environment and Water – Biodiversity, Conservation and Science, Parramatta, NSW, 2150, Australia; NSW Department of Climate Change, Energy, the Environment and Water – Biodiversity, Conservation and Science, Parramatta, NSW, 2150, Australia; Research Centre for Ecosystem Resilience, Botanic Gardens of Sydney, Sydney, NSW, 2000, Australia; Queensland Alliance for Agriculture and Food Innovation, The University of Queensland, St Lucia, QLD, 4067, Australia

**Keywords:** Threatened plants, policy, conservation, genetics, population genetics, budget, cost

## Abstract

The importance of conserving plant genetic diversity has been recognized since the 1980s, but genetic research tools for improving conservation remain largely absent from standard planning. Using an Australian case study framework of the New South Wales government’s Saving our Species (SoS) programme, we outline the costs and benefits associated with conducting genomic analysis within a conservation strategy to inform, for example, taxonomic resolution, targeted monitoring, translocations and *ex situ* collections. Despite a reported sentiment that costs are prohibitive, our study identified that where genetics reports have been provided (32 to date), the cost of genetic sampling, analysis and advice is <10 % of the total government investment (SoS programme) and will continue decreasing proportionally throughout the years as other management occurs. We identified that the largest government investment was for maintenance and monitoring actions. On-ground practitioner feedback from the reports identified that the main reason for requesting genetics was to inform translocation or *ex situ* collection. However, from the total of 269 plant species with translocation or *ex situ* conservation actions planned, 75.4 % still do not have provisions for genomics, suggesting that knowledge of the utility of this practice is low among practitioners. Responsive feedback also demonstrated that 90 % of respondents seek additional learning, and thus there is merit in providing future genomics-focused workshops.

## INTRODUCTION

The International Union for Conservation of Nature World Wildlife Fund Plant Conservation Programme (Hamann, 1985; Heywood, 2017) is one of the earliest large-scale plant conservation initiatives commenced in the mid-1980s. One of its targets (Target 3) aimed to promote *in situ* and *ex situ* conservation of plants (Hamann, 1985). Today an estimated 20–40 % of global plant species are threatened with extinction (Pimm and Raven, 2017, Lughada *et al.*, 2020) and plant conservation is led globally by both not-for-profit and government initiatives, with a focus on paired *in situ* and *ex situ* conservation actions (Heywood and Iriondo, 2003). In addition, most countries (176) have some form of framework law guiding conservation or protection of ecological or environmental assets (UN Environment Programme, 2019) such as biodiversity and natural resources. However, noticeably absent has been the uptake of genetic-based tools within conservation planning and actions (McMahon *et al.*, 2014). In this paper, we define genomics in agreement with McMahon *et al.* (2014), as analyses generated by next-generation sequencing (NGS) techniques such as whole-genome and transcriptome sequencing, data from reduced sequencing libraries and typing of single-nucleotide polymorphisms (SNPs). Conservation genomics, then, is the application of these methods specifically for conservation of biodiversity. For plants this usually means genomics applied to single threatened entities or vegetation communities.

Conserving plant genetic diversity is a dedicated action in the Global Strategy for Plant Conservation 2011–2020 (Objective II Target 5; Convention on Biological Diversity, 2011), will feature heavily in post-2020 targets (Convention on Biological Diversity, 2022) and has been an established field since the 1990s (Ouborg *et al.*, 2010). Despite the historic and ongoing emphasis of conserving genetic diversity, only recently has plant conservation genetics, mostly through the advent of genomic tools, begun to be incorporated into applied plant conservation planning strategies (Rossetto *et al.*, 2021; Hogg *et al.*, 2022). Further, plant conservation in general receives less prioritized funding and actions than animal-related conservation (Balding and Williams, 2016) and conservation genomics specifically is yet to be consistently embedded into government-led conservation planning. Where genomics is considered within government-led conservation, there is again an emphasis on applying methods to fauna (Pierson *et al.*, 2016). Despite strong practitioner recognition of the value of genetics to conservation (Frankham *et al.*, 2017; Taylor *et al.*, 2017), the uptake of genomics methods has traditionally been limited by a perception of high costs and analytical complexities (Taylor *et al.*, 2017; Taft *et al.*, 2020; Hogg *et al.*, 2022). Nonetheless, strategies for cost-effective application of plant conservation genomics have now advanced, with a standard workflow protocol (detailed in Rossetto *et al.*, 2021) capable of addressing numerous practical conservation decisions via a well-considered sampling design.

There are potential downstream implications of proceeding with conservation actions without consideration of genetics, such as misallocation of resources to taxonomically uncertain entities (e.g. *Banksia vincentia*; Wilson *et al.*, 2022), and overallocation of resources to unnecessary collection, replication, maintenance, *ex situ* storage and construction or enhancement of wild populations through the use of unsuitable lineages such as clones or siblings, which may compound threats through enhancing inbreeding and genetic erosion. The advent of reliable and increasingly cost-effective genomic tools (McMahon *et al.*, 2014), particularly large data sets of genome-wide SNPs (Sansaloni *et al.*, 2011; Brandies *et al.*, 2019) and more recently genome-wide association (GWA) methods (Uffelmann *et al.*, 2021), means that there is scope for inclusion of population genomics in the time-sensitive early phases of species conservation planning. The outstanding issues remain how to embed this approach within the current framework and ensure practitioners are equipped to interpret and apply results within the ecological knowledge base of their target organism (e.g. Frankham *et al.*, 2019).

### How is conservation structured in Australia?

In Australia, the circumscription of a species is based on taxonomic qualification using morphological features (i.e. morphological species concept). However, the inclusion of genetics coupled with morphological data has been previously suggested and used (Frankham *et al.*, 2012). Species are listed as threatened under Commonwealth and various state legislations using the Common Assessment Method, based on IUCN thresholds (DCCEEW, 2023). The Commonwealth legislation governing protecting listed entities is the *Environment Protection and Biodiversity Conservation Act 1999* (EPBC Act), which describes 2106 listed species, of which 1452 are plants (36 presumed extinct) (DCCEEW, 2024). In addition, 106 vegetation communities are listed as threatened (DCCEEW, 2024). Conservation actions for Commonwealth listed species have historically been managed by recovery plans, of which 43 % of plant-related plans noted genetic factors as risks for consideration (Pierson *et al.*, 2016). Accessing budget data for recovery and conservation actions is challenging, due to sparse accounting of species conservation expenditure and no legal requirements to allocate funds (Wintle *et al.*, 2019). Best estimates suggest that, as of 2018/19, the Australian funding allocated to threatened species conservation was AU$49.6 m (USD$38.1 m) federally and AU$122 m/year (USD$92 m/year), including all state and territory budgets (Wintle *et al.*, 2019). As of 2024, the Australian government allocates <0.1 % of the total federal budget to threatened species management (Biodiversity Council, 2024).

In New South Wales (NSW), species are listed under the *NSW Biodiversity Conservation Act 2016* (BC Act), which describes 1074 listed entities, of which 718 are plant, alga or fungi species and 31 are presumed extinct (NSW Threatened Species Committee, 2024). In addition, there are 111 listed vegetation communities. Under the BC Act 2016, listed entities must be managed by a dedicated threatened species management plan. This plan is entitled the NSW Saving our Species (SoS) programme, which has the overarching goal to secure NSW’s threatened species in the wild for the next 100 years (NSW Environment and Heritage, 2013).

The SoS programme provides specific management actions to secure listed entities that are guided by a prioritization protocol (Brazill-Boast *et al.*, 2018; Mayfield *et al.*, 2020). Of the 35 management actions that can be assigned as critical for conserving a species, the eight commonly applied to plant conservation include monitoring, land holder/land management engagement, fire planning, *ex situ* flora management, flora translocation, grazing management, targeted research and integrated weed control. The least commonly applied management action is grazing and fire management (as they are species- and site-specific). Notably absent, however, is an action dedicated to understanding species genetic diversity. Of the eight actions most relevant to plant conservation, genetics offers a substantial guiding benefit to four: *ex situ* conservation, *ex situ* flora management, flora translocation, and targeted research (across a range of relevant questions/topics). Population genetics can also be an essential pre-planning tool to help guide investment and conservation decision-making before entities are listed (e.g. to confirm species concept), and to assign management actions as well as support monitoring designs and interpretation of monitoring results (e.g. to guide genetically representative germplasm collection, translocation and monitoring). However, conservation genomics actions have only recently been integrated into NSW conservation planning and have so far only been on an *ad hoc* basis.

A key reportable outcome of the NSW SoS programme is transparency in return on investment (NSW Environment and Heritage, 2018). Investment refers to cash and in-kind contributions from both external stakeholders and internal SoS programme and department. Here we contextualize the financial investment required to undertake genomic analysis, relative to the requested and unanticipated applications of genomic data. To do this we reviewed the contribution of conservation genomics to the NSW SoS programme, in the context of (1) return on investment and (2) management actions that have occurred for the species. We also interviewed SoS project coordinators responsible for the delivery of management actions to gauge the value of genomics to their conservation efforts. We include three case studies where genomic studies have been critical for guiding management actions, either *a priori* or *ex post facto*.

## MATERIALS AND METHODS

### Genetic activities within Saving our Species plans

The Research Centre for Ecosystem Resilience (ReCER) is contracted by the NSW Department of Climate Change, Energy, the Environment and Water, to conduct genetic analysis of selected species from the SoS programme. The reports on genetic data produced by the ReCER team were cross-referenced with plant species listed in the NSW SoS database (SoS 4.9.0) to determine (1) how many listed species had actions that would benefit from genetics (e.g. translocation or *ex situ* germplasm collection) and (2) how many species of all those listed had genomic analysis planned or completed. These data were provided on request by the SoS programme.

Project Actions and total reported investment (including internal and external sources and both cash and in-kind contributions), for all species that have had genomic analysis, was manually extracted from species-specific Annual Report Cards stored in the New South Wales SoS database (SoS 4.9.0). The data are available on the Public Register of Saving our Species strategies (NSW Environment and Heritage, 2024). Manual extraction was required because Annual Report Cards are publicly available as PDF only and undertaking genetic assessment is not a listed Project Action. Instead, genetic analysis can be embedded as a line item or description within a larger allocated action (e.g. flora translocation). Years extracted were from commencement of SoS investment records (2015/16 financial year) to the most recent reported financial year (2022/23). The 2022/23 data were provided on request as it is currently unpublished. The SoS programme commenced in 2013 (NSW Environment and Heritage, 2013) in a development phase. The current SoS programme and framework commenced in 2016 with the adoption of the BC Act (2016). Although records of investment including and prior to 2015 were collected during development, they are not comparable with current data. Consequently investment prior to 2015 is not included in the reported figures.

Financial data for each species were recorded, per annum, as internal (SoS programme and NSW government internal) and external investment (i.e. support or cash provided as leveraged investment). Investment referred to both cash and in-kind contributions. For each financial year, investment was also broken down based on SoS actions. Action-level data were provided by data request from the SoS programme as it is not publicly available via Annual Report Cards. Where genetic budget investment was reported in the SoS database, these were extracted per annum. Where the SoS database did not report or record budget investment, the financial records of the ReCER were used. Data prior to 2011/12 were unavailable, and therefore the maximum duration of any conservation action is 12 years. Financial data were included only for species where the genetics had been completed and conservation advice reported as of the 2022/23 financial year. Conservation genomic studies for an additional 45 species are currently in progress, but because the reports had not been produced the costs had not consistently been included in SoS financial year expenditure reporting.

Results are presented with descriptive statistics. Costs are presented in total and per species for life of project, as well as per annum (mean ± standard error). These results are broken into internal (SoS and government) and external (other partner and stakeholder) investments. We intended to also quantify the in-kind and cash contributions for each species; however, these were not consistently recorded and have been manually calculated for case study species only (see Case studies in the Results section).

### Genomic methods

Among the 32 species for which ReCER has finalized conservation genomic studies, three case studies, involving the NSW listed and SoS managed threatened species *Banksia vincentia*, *Fontainea oraria* and *Prostanthera densa/P. marifolia*, are presented here to indicate the primary SoS actions associated with species management and exemplify how a genetic study can be implemented to assist with strategic management planning. The genetic studies are based on structured yet customizable workflows (Rossetto *et al.*, 2019, 2021), contingent on the conservation and management needs of the species projects. These studies demonstrate the effectiveness and versatility of genomic data in informing various conservation actions to enhance species management. The applications include confirmation of species concept, which provides justification for implementing relevant conservation actions, hybridization testing to identify the potential threat, and description of within- and between-population genetic structure to plan for *ex situ* and translocated populations. Conservation genomic studies conducted by ReCER are based on cost-effective yet highly informative genome-wide SNP data. The outputs of each study were documented in an official departmental report that was written in a concise and understandable format highlighting practical applications and circulated to the threatened species officer in charge of managing the species.

Specific details of case studies presented here have been published in Wilson *et al.* (2022) and Rossetto *et al.* (2000), where genomic methods are outlined. In brief, all species genomic analyses are based on mostly fresh leaf material sampled by the species project coordinator. Studies applied a high-throughput, restriction-based, reduced-representation genome sequencing method (proprietary diversity arrays technologies sequencing, DArTseq) to generate genomic data. The data analysis is not computationally intensive, applying well-established methodologies to infer relationships among individuals and populations. The data are useful for taxonomic resolution, and assessment of genetic health and diversity, which can contribute towards the design of genetically optimized *ex situ* collections and translocation designs.

### Practitioner feedback survey

Between August and December 2023, a survey aimed at assessing the utilization and value of the species-specific reports was distributed to the SoS project coordinators, responsible for managing the species of interest (Supplementary Data Table S1). Questions were predominantly multiple choice or ranked on a Likert scale (1–5) and invited respondents to give feedback about the readability of reports, most useful/relevant components, and areas for improvement. The survey also requested respondents to classify their motivation for requesting genomics, as well as any cost savings arising, with the question ‘Were there long-term cost savings as a result of including species/population genetic information?’ (see Supplementary Data Table S1 for response options). Respondents were not asked to quantify savings, as it was deemed too inconsistent for a practitioner feedback survey. Most questions were supported by free text and where relevant we used free-text survey responses to enrich the descriptive statistics. Open-ended questions were coded to themes using inductive content analysis (Thomas, 2006). We assessed trends based on the responses using mean Likert scores and by comparing percentages of binary (yes/no) and multiple-choice responses. Results were presented as a weighted average. The number of respondents completing each question varied and was reflected where sample size changed.

## RESULTS

### Genetic activities within Saving our Species

As of the 2022/23 financial year, 269 of the 897 plant species listed under the BC Act (30 %) have been assigned translocation- or *ex situ*-based SoS actions, which would directly benefit from genetic analysis. Of these 269 species, 75.4 % are not listed as part of ReCER work planning, 17.6 % have genetics planned and 6.9 % have had conservation genomics undertaken. There are 136 species with translocation-related SoS actions, of which 19 % have genetics planned or completed, and 172 plant species have assigned *ex situ*-related SoS actions, of which 23 % have genetics planned or completed.

**Fig. 1.**
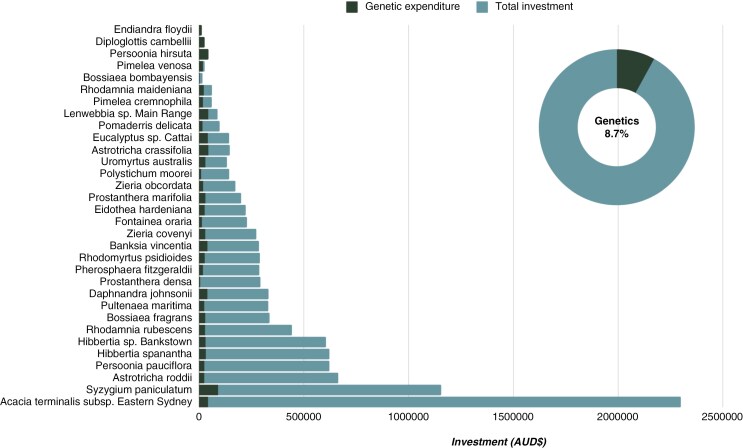
Stacked row chart presenting genetics proportion of total project investment for 32 plant species managed under the NSW Saving our Species programme. Reported investment is accurate date ranges 2015/16 financial year to 2022/23 financial year. Investment includes in-kind and cash contributions. Inset pie chart represents total investment of AUD$10 629 643, of which genetics contributes AUD$921 504 or 8.7 % of the total investment.

Thirty-two plant species managed under the SoS programme had genetics reports completed, as of December 2023. The total investment (cash and in-kind) for these species for the current framework (2015–16) to 2022–23 financial year close, is AUD$10 629 643, of which AUD$4 616 258 is from SoS investment and AUD$6 013 385 is from external investment (most commonly from local partner organizations and/or local government with species occurring on management sites). The 2023 average exchange rates were AUD/USD$0.664 and AUD$/€0.614. The total cost of genetics for these species is AUD$921 504, which equates to 8.7 % of the total expenditure across all projects (Fig. 1). Project duration varies and for the reporting period the average cost per project, per annum, is AUD$61 748 ± 12 775, which includes ongoing maintenance, monitoring and outreach activities. Genomics, by contrast, is most often a single event, with an average cost per species of AUD$28 797 ± 2807, which equates to AUD$7117 ± 1540 per annum. This cost includes sample processing, sequencing, analysis and interpretation as well as reporting and customized guidance. In addition to this, there are costs for sample collection, but often these costs can be minimized by coordinating sample collection with other activities such as maintenance or monitoring. Genetics is on average 19.3 ± 4.29 % of the total budget, varying from 1.8 % (*Acacia terminalis* subsp. Eastern Sydney) to 114 % (*P. hirsuta*, for which genetics occurred prior to project commencement in 2022/22). The most expensive report was produced in two parts and cost AUD$90 423; this was a research specific-project aimed at confirming the species concept for *Syzygium paniculatum* (Fig. 1) (Lu-Irving and Rossetto, 2021; Lu-Irving *et al.*, 2023). The high budget reflects intensive field sampling costs arising from a wide species distribution, as well as genome assembly and whole-genome resequencing of multiple samples to resolve taxonomic uncertainty. For other species, collection costs are minimal or absent when sample collection can co-occur with other regular site activities (e.g. surveys and maintenance). For example, the lowest genetics cost was AUD$5480 for *P. densa*, because co-analysis with other species could occur, and sample collection costs were included under the *P. marifolia* budget (Fig. 1; Case study 3). Two species, *Diploglottis campbelli* and *Endiandra floydii*, have had to date no recorded investment outside genetic analysis (Fig. 1).

Four non-listed entities (i.e. not SoS species) had co-analysis included as part of taxonomic resolution of the target species. This included three *Bossiaea*, as part of reconciling *B. bombayensis* and *B. fragrans* taxonomy (McMaster *et al.*, 2024), and one newly described *Endiandra* (Weber and Forster, 2021). The cost of the co-analysis was AUD$9500 for the three *Bossiaea* and AUD$13 500 for the *Endiandra*. These species now offer legacy genetic data that can be used to inform future conservation advice or actions, such as future threatened species listing, or ecological research, as required to protect these species.

**Fig. 2.**
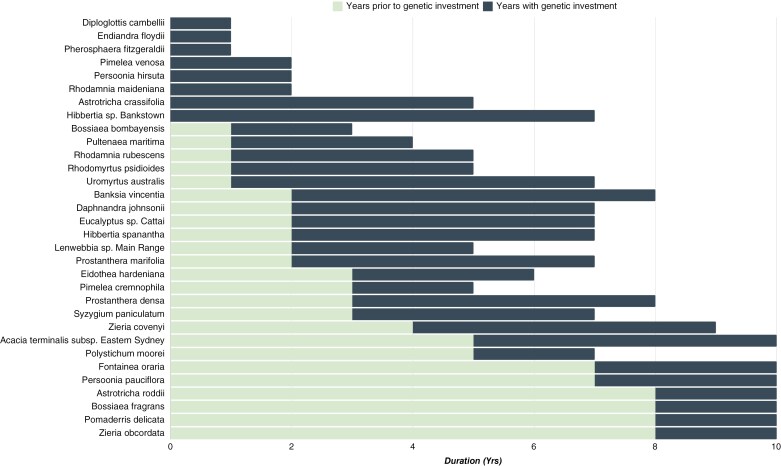
Duration of each species project (years) during the Saving our Species (SoS) programme, including years prior to and including genomic analysis as reported in the Annual Report Cards. Analysis commenced prior to or concurrent with project commencement for eight species and in the first year of the project for another four species. We note for some species work may have commenced prior to SoS and is not captured or represented in these data (e.g. *Fontainea oraria* conservation actions commenced in 2006 but the first recorded investment in genetics occurs in 2000)

Although SoS programme investment reporting commenced in 2015, legacy data are available from earlier conservation actions dating to the 2011/12 financial year. Eight species had projects commencing prior to 2015, the earliest recorded investment being in the 2013/14 financial year (official SoS commencement; NSW Environment and Heritage, 2013). We note, however, species conservation actions were often in place prior to SoS commencement (e.g. *F. oraria* conservation actions commenced in 2006 under previous legislation; D. Brown, species project coordinator, pers. comm.). Total project durations, including those that commenced prior to 2015, range from 1 to 10 years. The average project duration is 6.08 ± 0.57 years. The percentage of the project that occurred without genetics is, on average, 36.6 % of the total duration; however, there is large variation. For instance, eight species had genomic and other activity (e.g. maintenance, *ex situ* collection) investment commencing in parallel or previously (Fig. 2). Conversely, two species had 80 % of the invested years occurring prior to genetics. Consequently, many projects have progressed without consideration of the population relatedness, genetic health, or optimized germplasm collection. For one species, *F. oraria*, genomic assessment was included in early planning but due to the *ad hoc* nature of its inclusion, genetics was not formally costed or reported (Case study 2). The average time between contract and report provision was 1.25 ± 0.16 years, which includes sample collation, DNA extraction, analysis and reporting.

Conservation genomic reports are in preparation for an additional 45 species. This includes 22 orchids, for which a comprehensive taxonomic review is in development. The average cost for analysis of these, as yet, unreported species, is AUD$17 121.96 ± 395.91

The SoS species Annual Report Cards (NSW Environment and Heritage, 2024) indicate genetic studies were most often requested to inform germplasm collection or translocation planning. However the scope, reasons and intended application of genetic information were not always articulated by SoS officers managing the species and compiling the annual data. Detailed breakdowns of cost per action are included in the case studies.

The chosen case studies below represent different applications of conservation genomics, each highlighting the annual and total investments relative to priority actions, management actions, and potential future applications. Each of the case studies demonstrates application of customizable workflows, fully outlined by Rossetto *et al.* (2019, 2021). Detailed annual investment data are included in Supplementary Data Table S2.

### *Case study 1: taxonomic clarification of* Banksia vincentia

#### Background.

*Banksia vincentia* (Stimpson & P.H.Weston) was formally described in 2014 from a single population of fewer than 20 individuals at Vincentia in NSW (Stimpson *et al.*, 2014). This led to its critically endangered listing and the start of its SoS conservation programme. While it was distinguished from other species based on its habitat and semi-prostrate growth, it was also part of the then unresolved *B. spinulosa* complex, which included four other taxa (Stimpson *et al.*, 2014). Recovery actions included translocation and establishment of *ex situ* collections at various botanic gardens. However, in addition to uncertainty about taxonomy, there were concerns regarding the extent of hybridization, both *in situ* and *ex situ*, due to the species’ small population size and presence of co-occurring species.

#### Investment.

**Fig. 3.**
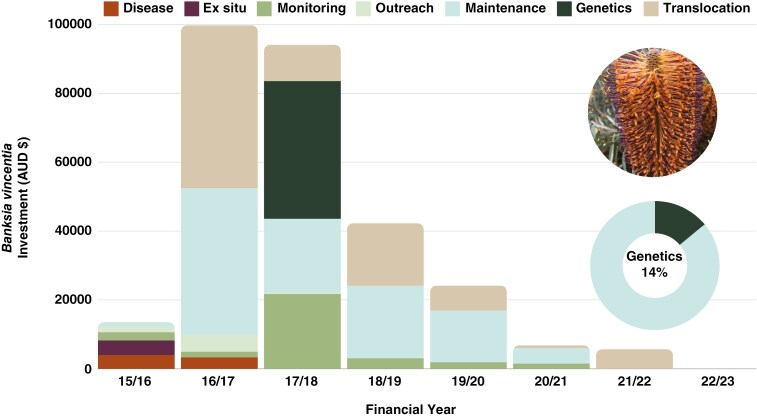
Stacked bar chart of the per annum investment in Saving our Species actions to conserve the putative species *Banksia vincentia*. Genetic analysis showed that *B. vincentia* was not a distinct species, and consequently investment gradually declined. As of 2022/23 no investment in conservation actions occurred. Inset pie chart represents total investment of AUD$286 213, of which genetics contributes AUD$40 020 or 14 % of the total investment.

Between the 2015/16 and 2022/23 financial years, a total of AUD$286 213 was invested in the conservation of *B. vincentia*. Most of the investment was spent on maintenance of a translocation site (AUD$105 700) in preparation for the translocation (AUD$89 699) that took place prior to the genetic study being commissioned (Fig. 3). The total investment on *B. vincentia* comprises AUD$181 748 in-kind (e.g. staff labour) and AUD$104 465 cash contributions, with genetic work costing AUD$40 020, or 14 % of the total budget (Fig. 3). From inception, genetics was included as a subsidiary action, embedded within ‘maintenance’ budgets (Supplementary Data Table S2).

#### Conservation genetics objectives.

To support conservation of *B. vincentia* a conservation genetic study was conducted to:

- Investigate its species status, and if verified as a distinct species, assess its genetic health (i.e. amount of genetic diversity).
- Determine the level of hybridization between *B. vincentia* and co-occurring congener species.
- Examine representativeness of the species’ genetic diversity (compared with wild populations) in dedicated *ex situ* collections that included putative seedlings and clones.
- Determine optimal material to be used in eventual translocation scenarios using *in situ* and *ex situ* material.

#### Outcomes and relevance.

Detailed methods and results are provided in Wilson *et al.* (2019, 2022). Results indicated that *B. vincentia* is not a distinct species and that most *ex situ*-propagated seeds derived from *in situ B. vincentia* plants were the product of hybridization with co-distributed *B. ericifolia* and *B. spinulosa* plants. Cryptic hybrids were detected *in situ* at the Vincentia site.

The findings provide compelling evidence to warrant the removal of *B. vincentia* from the threatened species lists. This significant change will allow redirection of resources, funding, and effort towards conservation of other threatened species. The comprehensive study involving other species also provides additional legacy results that resolve relationships within the *B. spinulosa* complex, in that the taxonomic statuses of other, non-target species were clarified.

Prior to the genetic research, a total of AUD$207 301 was invested in conservation actions. Following the release of the genetic report (Wilson *et al.*, 2019), investment (both cash and in-kind) gradually declined (Fig. 3), totalling AUD$78 911 over the following 5 financial years. Ongoing expenditures were primarily for the maintenance of the *ex situ* collection and monitoring the wild population while the taxon is in the process of being formally delisted (as yet incomplete). As of 2022/23 no actions were required due to ‘taxonomic uncertainty’. It is difficult to predict future savings from the application of genomics; however, it is certain that a large portion of the initial AUD$207 301 could have been saved and allocated to other species if genomics were applied prior to actions. In addition, an average of AUD$61 748 ± 12 775 is spent per annum on species analysed in the data set. Ideally, this allocation would be reallocated, theoretically, in perpetuity.

### *Case study 2: genetic rescue of* Fontainea oraria

#### Background.

*Fontainea oraria* is a critically endangered littoral rainforest species, known from two sites near Lennox Head, NSW. The wild population numbered only 10 adult plants and 52 seedlings in 2016 (Brown *et al.*, 2016). It is mainly dioecious, with plants having predominantly male or female flowers, but male plants have been observed to occasionally produce female flowers. Early genetic studies that commenced prior to SoS confirmed *F. oraria* as a unique species, disproved clonality and identified unequal representation of parent plants in seedling cohorts (Rossetto *et al.*, 2000). This early study also initiated *ex situ* conservation collection and translocation. Genomics were commissioned within SoS to assist with monitoring translocation success and customization of the *ex situ* collections (Yap and Rossetto, 2020, published in Rossetto *et al.*, 2023).

#### Investment.

**Fig. 4.**
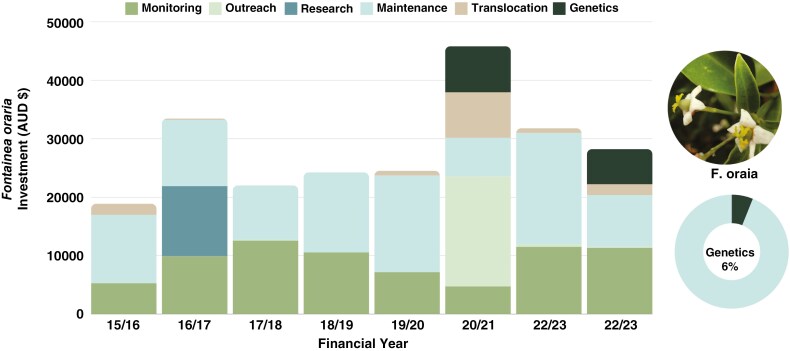
Stacked bar chart of the per annum investment in Saving our Species (SoS) actions for genetic rescue of *Fontainea oraria*. Earlier investment included initial genetic reporting as part of preparing an *ex situ* collection and translocation but is not included in the SoS investment, as this investment was recorded on an *ad hoc* basis. Inset pie chart represents total investment of AUD$228 920, of which genetics contributes AUD$18 062 or 6 % of the total investment.

A total of AUD$228 920 was invested in *F. oraria* conservation from 2015/16 to 2022/23, and prior to this period genetics was conducted to verify its species status and the level of genetic diversity that was present (Rossetto *et al*., 2000). Total documented investment comprised AUD$197 470 cash and AUD$25 280 in kind (in 2021/22 AUD$68 132 was not attributed to either cash or in kind), with genetics costing AUD$13 862, or 6 % of the total investment (Fig. 4). The largest allocations were for site maintenance (AUD$97 023) and monitoring (AUD$73 003) (Fig. 4). Genetics was included as a subsidiary action, embedded within ‘translocation’ and species ecological ‘research’ actions (Supplementary Data Table S2).

#### Conservation genetics objectives.

This study sought to quantify the success of the translocation programme through sampling wild, translocated and *ex situ* populations with the following aims:

- Determine the parentage of seedlings recruited from the translocations.
- Determine if translocated population genetic diversity represented wild population diversity.
- Identify if the level of contribution by each reproductive adult to the seedling cohort is higher for the translocation sites than the wild site.
- Suggest improvements to existing translocated populations to ensure long-term viability.
- Detect potential mislabelling of *ex situ* plants used in translocation.

#### Outcomes and relevance

- Genetic diversity of translocated sites was representative of wild population.
- Parentage was identified for all seedlings (except one, where only one parent could be identified).
- Some translocation sites had unique parentage not represented in the wild progeny.
- Seedling genomic diversity was greater than the ten extant adults, demonstrating that sexual recombination was producing novel and unique genotypes.
- Under-represented genotypes (father/mother) plants were identified in the initial recruitment events.
- Misidentification of plant tags was detected.
- For translocations, 231 specimens were planted across 22 locations between 2010 and 2023. An estimated 5629 seedlings were recorded in 2023.
- Monitoring of genetic rescue success, determined through ongoing recruitment and survival of genetically diverse progeny.

These collective analyses, particularly the application of genomics to monitoring, provide a working proof of the genetic rescue concept, where populations created through translocation are not only viable but produce novel genotypes not seen in wild plants. Further genetic monitoring or screening can be used to identify under-represented genotypes and facilitate targeted crossing, as required. The application of genetics to screening *ex situ* collection also presents a retroactive method to reduce downstream complications of mislabelling.

The genetic results demonstrate that current efforts in genetic rescue are successful, and species recovery is on track. An additional translocation site is planned on secure National Park & Wildlife Services (NPWS) tenure which will use seedlings produced from existing translocations that have been identified as representing unique genetic combinations, and those with fewer representatives at the existing translocation sites. Ongoing field monitoring shows that wild and translocated plants are thriving and display recruitment, although natural seedling attrition is anticipated. To reduce impacts on wild plants, seed for use in research and *ex situ* conservation is sourced from translocated plants. As part of long-term species management, strategic planning will include genomic surveys to identify the need for interpopulation crossing, track parental contribution, minimize genetic erosion and maximize population diversity. Resource savings from the use of genomics can in this instance only be inferred and include time-effective labour for *ex situ* collection and translocation design, as well as reduction in downstream implications of mislabelling (which was detected early using genomics). Genomics will also optimize time spent on future population mixing, and potential hand pollination to ensure under-represented genotypes are retained.

### *Case study 3: untangling* Prostanthera species *confusion*

#### Background.

*Prostanthera densa* A.A. Ham. and *P. marifolia* R.Br. were frequently confused due to their similar appearances, with their distinction based on vegetative characteristics. *Prostanthera marifolia* is a prostrate shrub with small leaves and was suspected to have some level of clonality, whereas *P. densa* is a robust upright shrub with larger leaves appearing not to be clonal. There were concerns about both species’ decline due to limited information about reproduction and viable seed rarely being observed. The distribution range of each species did not assist with clearing up any taxonomic confusion; even though they are currently non-sympatric, it was suspected that historically both species would have had overlapping distributions (Conn *et al.*, 2013).

A preliminary molecular phylogenetic analysis of the genus that used both maternally inherited chloroplast and nuclear DNA markers (Wilson *et al.*, 2012) confirmed the two species were closely related. Conn *et al.* (2013) confirmed distinction between populations, again using paired chloroplast and nuclear DNA methods. Neither study was able to fully resolve species boundaries, the latter concluding that high-resolution sampling and molecular markers would be required for a better test of species boundaries, diversity and evolutionary history.

#### Investment.

**Fig. 5.**
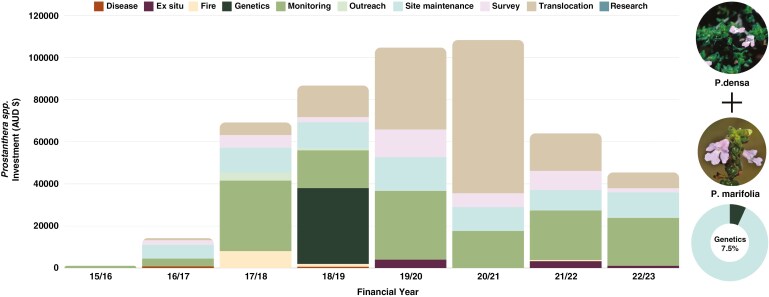
Stacked bar chart of the per annum investment in Saving our Species actions for resolution of *P. densa* and *P. marifolia*. Single investment in genetics confirmed taxonomy and status of *ex situ* collections; however, without in-field tagging of plants, customized translocation advice could not be provided. Nonetheless, translocations for *P. densa* occurred at a total cost of AUD$116 485. Inset pie chart represents total investment of AUD$494 070, of which genetics contributes AUD$36 045 or 7.3 % of the total investment.

A total of AUD$494 070 has been spent to date on the conservation programme of *P. densa* and *P. marifolia* between the financial years 2015/16 and 2022/23. Investments comprised AUD$291 863 cash and AUD$202 208 in-kind contributions. An amount of AUD$36 045, or 7.3 % of the total investment, was allocated for genetics in the financial year 2018/19. The largest allocations of the investment took place prior to genetic study and were for translocation-related actions (AUD$159 273) as well as monitoring (AUD$153 053) (Fig. 5). Genetics was included as a subsidiary action, embedded within ‘translocation’ and ‘monitoring’ actions (Supplementary Data Table S2).

#### Conservation genetics objectives.

A report produced by Yap *et al.* (2020) aimed to address many of the uncertainties between these two putative species, including:

- Test the species concept of *P. densa* and *P. marifolia* using population genetics (DArTseq).
- Assess the extent of clonality, kinship and genetic diversity within and between wild populations.
- Assess diversity and genetic provenance of the *ex situ P. densa* and *P. marifolia* collections.
- Inform management strategies as needed.

#### Outcomes and relevance

- *Prostanthera densa* and *P. marifolia* are related but genetically distinct species based on population genetic divergence.
- A *P. densa* site with intermediate morphological features appears genetically intermediate between pure *P. densa* and *P. marifolia*, and possibly represents a morphological extreme of either species, or a vestige of a contact zone from an ancestral hybridization (Yap *et al.*, 2000).
- *Prostanthera densa* is characterized by strong population structure (differentiation), and as such its northern and southern groups can be managed as separate entities. This would not increase costs as both sites require monitoring and some maintenance.
- Options for genetic rescue, in the case of population decline, can consider mixing the two groups, and the risk of outbreeding depression will likely be less than that of inbreeding depression (Frankham *et al.*, 2011; Ralls *et al.*, 2017).
- Both species have low within-site diversity, likely because of no gene flow between populations, and therefore have relatively high levels of inbreeding.
- Contrary to expectation, clonality was not detected in *P. marifolia*. Clonality was detected in *P. densa* despite no prior suspicions and, given no detection of a rhizomatous habit, may be the result of apomixis (i.e. asexually produced seed).
- The *ex situ* collections of *P. densa* and *P. marifolia* are unrepresentative of genetic diversity in the wild, consisting of only one genet for each species. This indicates comprehensive recollection for genetically diverse *ex situ* collections.
- A selection of a minimum of 10 genets (northern) and 18 genets (southern) is required to maximize genetic diversity in *ex situ* collections [i.e. 95 % of (known) genetic diversity captured].

The confirmation of two distinct species has reduced potential misallocation of resources, such as if these two *Prostanthera* were not distinct species. Due to simplified sample collection, and untagged wild plants for *P. densa*, this study represents a missed opportunity to simultaneously use data to inform genetically optimized translocations and identifies a need to establish rigorous data collection methods prior to undertaking genomic analysis. Systematic species sampling, including tagging of wild plants for reliable data integrity, is required before planning genetically optimized *ex situ* collections or translocations.

No site-level conservation action has occurred for *P. marifolia* as wild recruitment occurred after an intentionally introduced ecological burn. However, future genomic screening of seedlings is planned and will complement planned establishment of an *ex situ* collection, which has begun with propagation trials and a draft translocation plan.

For *P. densa* the original intent of the study was to confirm its distinction as a species, hence sampling rates were low (as well as the budget). However, site augmentation translocations did occur (total expense AUD$116 485), without the inclusion of comprehensive genetic advice. This presents a potential problem for long-term population viability given the knowledge of clonality and uncertain within-population diversity. Although these translocations had varied success (some with reproduction and others resulting in plant death), the low initial sampling has meant that there remains a need for subsequent sampling of wild, *ex situ* and translocated populations. Tagging of plants has occurred. Re-collection of samples is planned, with the aim of undertaking a more comprehensive study that will optimize future translocations and inform current monitoring.

### Conservation genomics feedback

**Fig. 6.**
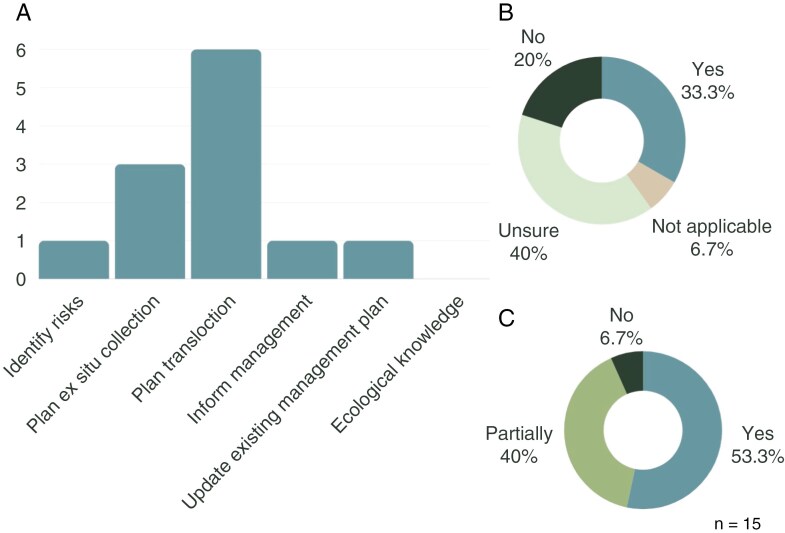
Survey respondent feedback to the questions (A) ‘Why was the report required?’, (B) ‘Did the genetics report generate new knowledge?’, and (C) ‘Did the content of the genetics report generate cost savings?’. Sample size for questions (B) and (C) was 15, and for question (A) sample size was 12, because three respondents declined to answer this question. Also, for question (A) no respondents identified ‘ecological knowledge’ as a motivator for requesting the genetics report.

We collected survey responses from 15 SoS conservation project officers, representing a total of 26 of the 32 plant taxa for which conservation genetics reports have been prepared (one project officer can represent multiple species). Responses were aggregated per officer and not per species-specific report. Most respondents requested genetics as part of applied management (translocation and *ex situ* collection), and no respondents identify genetics as a method to inform species ecological process (e.g. breeding system(s) such as clonality or apomixis) identify ecological knowledge (Fig. 6)

Over 90 % of respondents suggested the genetics supplied new or partially new knowledge. Although 40 % of respondents were uncertain about the contribution of genetics to cost saving, 33 % identified cost savings generated from applying genetically informed data to management. How these cost savings were surmised was not quantified.

Respondents found the reports readable and useful (Table 1), with the most ‘useful’ component being a section dedicated to management recommendations. Sixty per cent of respondents commented, however, that they would like additional assistance interpreting and applying reports and 93 % indicated interest in a dedicated workshop focused on plant genomics. This suggests a desire for additional skills. Six respondents provided detailed recommendations for improvements; most commonly these focused on simplified explanations to accompany genetic terms/the inclusion of a glossary and greater focus on application of data to management or specifics of how to use genomic data to revise a species taxon, actions that have been considered in future reports. Free-text contributions also identified that practitioners recognize the value of genetics to applied management; however, there was some initial concern about collection methods/skills, and that the time between commissioning and reporting can be prohibitive (Text Box 1).

**Table 1.** Summarized responses from Saving our Species conservation project officers (*n* = 15) about the accessibility and relevance of genetics reports to conservation planning. Averages were calculated from Likert 1–5 scores, where 1 represents lowest and 5 highest.

Question	Response (weighted average)
Readability of report	4.53
Usefulness of the genomic information contained in the report/s?	4.6
Ease of ability to interpret the results and use the information?	4.3

Text Box 1. **Practitioner responses to the genetics reports provided as part of conservation planning for threatened plant species within the NSW Saving our Species programme**

‘I have been inspired to consider using genetic data in other projects, given the simplicity of collecting it. The team may need expansion as I can see the importance and popularity of the method growing! The (understandable) time it takes to deliver the results may be prohibitive for some projects’.

‘It is a strong basis for the start of management of each species so would have allowed for management actions such as *ex situ* collections to be developed quicker’.

## DISCUSSION

As the rate of species loss continues, prioritization of conservation management actions is essential to maximize success, being mindful of resource and time constraints. Conservation genetics has long been cited as a valuable planning tool within targeted species conservation actions, by researchers (Ouberg *et al.*, 2010; Frankham *et al.*, 2019; Rossetto *et al*., 2021), practitioners (Taylor *et al.*, 2017), international (e.g. CBD, 2011) and, occasionally, national policy (Wintle *et al.*, 2019). Despite this, there remains a conservation genetics gap between conceptual support and the physical application of genomics to conservation planning and outcomes (Klütsch *et al.*, 2021), with a bias where it occurs, towards northern hemisphere fauna (Tkach and Watson, 2023). This conservation genetics gap is attributed to cost (Taylor *et al*., 2017), a lack of meaningful linkages between research, management and policy (Britt *et al.*, 2018), and a shortage of skilled personnel to analyse, interpret and apply results effectively in conservation contexts (Cook and Sgrò, 2018; Taft *et al.*, 2020).

In our study we reviewed the current application of genomics to the largest threatened plant species conservation management in NSW, the Saving Our Species programme (Brazill-Boast *et al*., 2018). To date genomics has been applied to 32 species, with an additional 45 planned. Where genomics has been applied, a report, with well-designed sampling, has been able to address a range of questions, whilst simultaneously providing direction for future applied management decisions. The areas most often addressed include confirmation of species concept and identification of population genetic health, including inbreeding and kinship, hybridization testing and optimization of diversity for *ex situ* and translocation planning. For species where translocation has taken place, genetic monitoring is applied to determine the efficacy of the established population, such as the level of genetic rescue in the translocated population (e.g. Case study 2, *Fontainea oraria*). To date genetics has been under-utilized as a tool to help prioritize costly maintenance and monitoring activities. We identified that nearly three-quarters of the SoS managed plant species with listed actions benefiting from genetics (e.g. *ex situ* collection, translocation) do not currently have genetics included as a planned action. The general absence of population genetics for fundamental planning presents an opportunity to embed genetics as a tool in future species management.

### Costs

Despite a reported sentiment that costs are prohibitive, and thus limiting the uptake of genomics for conservation planning (Taylor *et al.*, 2017), our study identified that, where reports have been provided, the cost of genetic sampling, analysis and advice is less than 10 % of the total SoS investment. These costs will continue decreasing proportionally throughout the years as other management occurs. Costs reduced where co-analysis occurred and conversely costs increased under large-scale field sampling or where whole-genome sequencing is required. Per annum, the average total investment in species conservation is AUD$61 748 ± 12 775 per species, and although genetics is a small proportion of the total budget, it does present a large portion of the annual expenditure at an average of AUD$28 797 ± 2807 per species. Often genetic costs are associated with a single sampling and reporting event; however, in some instances genetics can be applied strategically to inform monitoring and efficacy of translocations (e.g. *F*_1_ generation parentage or to gauge the success of genetic rescue action). We suggest that conservation planning should allocate dedicated funding at the commencement of projects to undertake critical genomic knowledge gathering, to inform and prioritize future planning, as opposed to the later inclusion of genetics into already established conservation programmes. Based on the summary data presented here, the inclusion of a small allocation to genetics at the start of the conservation activities is likely to guide conservation activities more accurately and consequently may save resources in the longer term. As genetic pipelines are streamlined, these costs will further decline, as is already evidenced by the fact that the average cost for an additional 45 species with analysis pending has already decreased (AUD$17 121.96 ± 395.91).

### Application of genomics to conservation actions

The benefits of genetics will be greatest when implemented prior to project commencement, or as a strategic part of ongoing monitoring. Implementing genetics retroactively can limit the much broader application of genomic data to general conservation planning and targeted resourcing. For instance, in the case of *B. vincentia* a total of AUD$207 201 was invested unnecessarily, from external sources, prior to the undertaking of genetics. A well-designed conservation genomics pipeline as outlined by Rossetto *et al.* (2021) can, in addition to addressing the target management question, also confirm or disprove fundamental taxonomical and ecological questions. The resolution of taxonomy and ecological factors, such as the breeding system and clonality, population fragmentation, inbreeding and kinship, will not only help plan translocation or *ex situ* collections, but help prioritize actions at a species and population level.

Practitioners identified that their primary motivation for requesting genetics was to inform a specific management action, usually to do with translocation planning and establishing or refining *ex situ* collections. Whilst practitioners acknowledged that the reports met their needs, to a high standard, we note that a large proportion of respondents (60 %) identified a desire for additional assistance interpreting or applying the outcomes (e.g. one practitioner requested support for how genetics could be used to recommend species taxonomic revisions). This underscores the need for additional communication, training and workshops to educate conservation officers in effectively utilizing genomic-based information with confidence, as well as integrating genomics into species listing assessments. Training initiatives, we expect, will empower practitioners to leverage genetic insights more effectively in conservation decision-making processes, ultimately enhancing conservation outcomes.

### Limitations

The cost of genomics has historically been identified as prohibitive (Taylor *et al.*, 2017; Taft *et al.*, 2020), and in our analysis if viewed as a single cost relative to annual budget may be perceived as a barrier. We argue, however, that this barrier can readily be overcome by front-loading project investment to undertake species-relevant ecological and genetic investigation (i.e. address gaps in knowledge) as part of informing future management, and planning for any subsequent genetic monitoring (e.g. as part of long-term translocation/genetic rescue actions). Where viewed as a single investment, relative to total project costs, genomics will become an increasingly small contribution through the passage of time with further enhancement of genomic techniques.

Our survey results mirror previous studies (Taylor *et al.*, 2017; Cook and Sgrò, 2018; Taft *et al.*, 2020), which identify a strong desire for collaboration with geneticists, and, based on the >90 % of respondents wishing for additional workshops, an interest in training and upskilling in ability to interpret and apply results to management. Addressing this skills gap is important too, to ensure that applied management occurs within the framework of a species biology and ecology. Limitations to achieving this collaboration and upskilling are the availability of geneticists to collaborate in compiling and producing reports rapidly. We identified the duration of time between commissioning and production of a report to be, on average, 1.25 ± 0.16 years, the main portion of the time being usually spent on sampling plant material. Practitioner feedback suggests that this delay can also be perceived as prohibitive (Text Box 1).

Further, the production of co-analysis data, whilst cost- and resource-effective, is limited by the ability of end users to access the data. A formalized global platform to retain data would benefit conservation endeavours, particularly those acting outside the NSW SoS programme.

## Conclusions

Genetics has moved from a novel, but costly, exploratory action to being a critical and standard tool to support ecological research and to prioritize and streamline management efforts. Genetics should no longer be considered a tool exclusively practised within the research sector but rather an *a priori* examination, embedded within conservation planning and prioritization frameworks. To achieve this, we propose the following:

1. Embedding of genetic diversity conservation within environmental legislation and policy.
2. Standardized management plan development, with higher investment in the early project years to conduct fundamental genetic and ecological rescue.
3. Population-scale genetic sampling to inform and help prioritize future high-investment recurrent actions such as maintenance and monitoring.
4. Use of population-scale genetic data to prepare genetically optimized *ex situ* collection and translocation plans that complement ecological knowledge.
5. Conducting co-collection and analysis where available to reduce resourcing requirements and costs.
6. Planning for genetic monitoring to complement standard monitoring at regular intervals to help direct and prioritize efforts. Additionally, where translocations have occurred, actively manage to optimize the genetic diversity, if required.

## SUPPLEMENTARY DATA

Supplementary data are available at *Annals of Botany* online and consist of the following.

Table S1: feedback survey distributed to SoS project officers, including questions and response format. Table S2: detailed annual investment, per activity, for case study species. Genetic studies were not a separate action, and consequently investment was allocated to differing actions. For *B. vincentia* genetics was embedded in the maintenance budget, for *F. oraria* genetics was embedded within translocation and species ecology, for *P. densa* genetics was included in monitoring, and for *P. marifolia* genetics was included as a translocation action.

mcae222_suppl_Supplementary_Tables_S1-S2

## Data Availability

Financial data used in this publication are available publicly from the New South Wales Saving Our Species Annual Report Cards stored in the New South Wales SoS database (SoS 4.9.0): https://www.environment.nsw.gov.au/topics/animals-and-plants/threatened-species/saving-our-species-program/saving-our-species-reports. Data were extracted from species-specific report cards. Raw data used in case study budgets are provided in the Supplementary material.
