# Supplementary material for: Conservation genomics within government led conservation planning: an Australian case study exploring cost and benefit for threatened flora
Source: Ann Bot. 2025 Jul 14;135(6):1229–42. doi: 10.1093/aob/mcae222 (PMC12259528; doi:10.1093/aob/mcae222)
Supplement: mcae222_suppl_Supplementary_Tables_S1-S2 [file mcae222_suppl_supplementary_tables_s1-s2.docx]

# Supplementary Material

Table S1: Feedback survey distributed to SoS project officers, including questions and response format. Likert scale 1-5 where 1 is the lowest rank and 5 represents the highest.

| **Question number** | **Question** | **Response format** |
| --- | --- | --- |
| Question 1 | Species name/s (include all where multiple reports received) | Free text |
| Question 2 | Please rank the readability of the report/s content | Likert Scale 1-5 |
| Question 3 | Please rank the usefulness of the genomic information contained in the report/s? | Likert Scale 1-5 |
| Question 4 | Please rate how easily you were able to interpret the results and use the information? | Likert Scale 1-5 |
| Question 5 | Would you like additional assistance, beyond what is already provided?  If so, what? | Binary Yes/No  Free text |
| Question 6 | What part of the report/s did you find most useful? | Free text |
| Question 7 | What information could be added or removed to improve the report/s? | Free text |
| Question 8 | Are there other stakeholders who do/should receive these reports? Who? | Free text |
| Question 10 | Did the report generate or change management outcomes? (If yes or partial please elaborate) | Select  **Yes**- on ground/planned actions were developed as a result of the report  **Partially**- the report informed an existing strategy, but some actions were already in place  **No**- the report contents was not relevant to on ground management or planning actions  **No-** the strategy was developed before the report was received and updates aren't planned  Free text |
| Question 11 | Why did you require this report? | Select  Identify risks to population/s  Plan targeted *ex situ* collection  Plan targeted translocation  Inform development of new management plan/recovery strategy  Update existing management plan  No specific reason. Part of assembling species ecological knowledge |
| Question 12 | If you had genetic data before you started this project, was it/would it have been relevant? How? | Free text |
| Question 13 | Were there long-term cost savings as a result of including species/population genetic information? | Select  Yes  Unsure  No  NA |
| Question 14 | Would you be interested in attending a conservation genomics workshop conducted by ReCER? | Select  Yes  No |
| Question 15 | Do you have any additional comments | Free text |

Table S2: Detailed annual investment, per Activity, for Casestudy species. Genetic studies were not a separate Action, and consequently investment was allocated to differing Actions. For B. vincentia genetics was embedded in Maintenance budget, for F. oraria genetics embedded within translocation and species ecology, for P. densa genetics was included in monitoring and for P. marifolia genetics was included as a translocation Action.

| **Species/**  **Actions** | **Financial Year** | | | | | | | | | | **Total Expenditure ($AUD)** |
| --- | --- | --- | --- | --- | --- | --- | --- | --- | --- | --- | --- |
|  | **13/14** | **14/15** | **15/16** | **16/17** | **17/18** | **18/19** | **19/20** | **20/21** | **21/22** | **22/23** |  |
| ***Banksia vincentia*** |  |  | **13495.51** | **99733.4** | **94072** | **42250** | **24132** | **6832** | **5697.84** |  | **286212.75** |
| Disease |  |  | 4018.6 | 3362 |  |  |  |  |  |  | 7380.6 |
| *Ex situ* |  |  | 4250 |  |  |  |  |  |  |  | 4250 |
| Genetics |  |  |  |  | 40020 |  |  |  |  |  | 40020 |
| Monitoring |  |  | 2336 | 1614 | 21749.6 | 3100 | 1932 | 1532 |  |  | 32263.6 |
| Outreach |  |  | 1600 | 4899.5 | 400 |  |  |  |  |  | 6899.5 |
| Site maintenance |  |  | 400 | 21200 |  |  |  |  |  |  | 21600 |
| Translocation |  |  | 90.91 | 47257.9 | 10502.4 | 18150 | 7200 | 800 | 5697.84 |  | 89699.05 |
| Maintenance |  |  | 800 | 21400 | 21400 | 21000 | 15000 | 4500 |  |  | 84100 |
| ***Fontainea oraria*** | **44836** | **17126** | **18876** | **33456** | **22014** | **24238.03** | **24514.32** | **45810.58** | **31799.66** | **28211** | **290881.59** |
| Genetics |  |  |  |  |  |  |  | 7862 |  | 6000 | 13862 |
| Monitoring |  |  | 5276 | 9900 | 12570 | 10550.03 | 7170.32 | 4732.27 | 11490.01 | 11315 | 73003.63 |
| Outreach |  |  |  |  | 180 |  |  | 18864.31 | 460 | 200 | 19704.31 |
| Research |  |  |  | 12000 |  |  |  |  |  |  | 12000 |
| Translocation |  |  | 1900 | 200 |  |  | 800 | 7778 | 800 | 1848 | 13326 |
| Maintenance |  |  | 11700 | 11356 | 9264 | 13688 | 16544 | 6574 | 19049.65 | 8848 | 97023.65 |
| Not recorded | 44836 | 17126 |  |  |  |  |  |  |  |  | 61962 |
| ***Prostanthera densa*** |  |  | **1063.8** |  | **33154** | **41216** | **67728** | **78500** | **38105** | **33436.43** | **293203.23** |
| *Ex situ* |  |  |  |  |  |  | 4000 |  |  |  | 4000 |
| Fire |  |  |  |  | 700 | 700 |  |  |  |  | 1400 |
| Genetics |  |  |  |  |  | 5480 |  |  |  |  | 5480 |
| Monitoring |  |  | 1063.8 |  | 14297 | 15300 | 24886 | 17500 | 17725 | 22681.93 | 113453.73 |
| Outreach |  |  |  |  | 3275 | 875 | 175 |  |  | 250 | 4575 |
| Survey |  |  |  |  | 4396 | 2400 | 3250 | 6500 | 9000 | 2000 | 27546 |
| Translocation |  |  |  |  | 5000 | 10600 | 29000 | 53000 | 11380 | 7504.5 | 116484.5 |
| Maintenance |  |  |  |  | 5486 | 5861 | 6417 | 1500 |  | 1000 | 20264 |
| ***Prostanthera marifolia*** |  |  |  | **14255** | **36050** | **45592.15** | **37097.5** | **29925** | **25923** | **12025** | **200867.65** |
| Disease |  |  |  | 780 |  | 565 |  |  |  |  | 1345 |
| *Ex situ* |  |  |  |  |  |  |  |  | 3290 | 1000 | 4290 |
| Fire |  |  |  |  | 7350 | 700 |  |  | 525 |  | 8575 |
| Genetics |  |  |  |  |  | 30565 |  |  |  |  | 30565 |
| Monitoring |  |  |  | 3700 | 19250 | 2712.15 | 7787.5 | 175 | 5800 | 175 | 39599.65 |
| Outreach |  |  |  |  | 350 |  |  |  |  |  | 350 |
| Site maintenance |  |  |  |  |  |  |  | 350 |  | 500 | 850 |
| Survey |  |  |  | 2175 | 1500 |  | 9830 |  |  |  | 13505 |
| Translocation |  |  |  | 1000 | 1000 | 4450 | 9980 | 19900 | 6458 |  | 42788 |
| Maintenance |  |  |  | 6600 | 6600 | 6600 | 9500 | 9500 | 9850 | 10350 | 59000 |
